# Proteomic Analysis Reveals That Metabolic Flows Affect the Susceptibility of *Aeromonas hydrophila* to Antibiotics

**DOI:** 10.1038/srep39413

**Published:** 2016-12-19

**Authors:** Zujie Yao, Wanxin Li, Yi Lin, Qian Wu, Feifei Yu, Wenxiong Lin, Xiangmin Lin

**Affiliations:** 1 Fujian Provincial Key Laboratory of Agroecological Processing and Safety Monitoring, College of Life Sciences, Fujian Agriculture and Forestry University, Fuzhou 350002, PR China; 2 Key Laboratory of Crop Ecology and Molecular Physiology of Fujian Universities, Fujian Agriculture and Forestry University, Fuzhou 350002, PR China; 3 Nanping Enter-Exit Inspection and Quarantine Bureau, Nanping 353000, PR China

## Abstract

The overuse of antibiotics results in the development of antibiotic resistance and limits the useful life of these drugs in fighting bacteria, including *Aeromonas hydrophila*, a well-known opportunistic pathogen that causes serious infections in fish and other animals. In this study, we investigated the adaptive resistance mechanism in *A. hydrophila* by multiple proteomic methods. Dimethyl labeling and label-free methods were performed to compare the differential expression of proteins in response to various doses of oxytetracycline (OXY). The results point to the conclusions that, in response to OXY stress, translational processes increase the abundance of these proteins whereas largely central metabolic pathways decrease their abundance. To confirm our hypothesis, various exogenous metabolites were compounded with OXY, and the resulting survival capabilities were measured. Results show that 7 metabolites (malic acid, serine, methionine, etc.) significantly decreased the survival capabilities of *A. hydrophila* in the presence of OXY, whereas 4 metabolites (arginine, lysine, tyrosine, etc.) did the opposite. Further investigation suggests that a compound comprising exogenous metabolites in combination with various antibiotics could have a significant bactericidal effect and might come into widespread use, especially together with tetracycline antibiotics. These findings may provide new clues to the antimicrobial treatment of *A. hydrophila* infection.

It is well known that owing to the abuse or misuse of antibiotics over the past 90 years, antibiotic resistance has become a serious healthcare problem. Furthermore, some 80% of antibiotics are used to treat livestock and other farm animals, leading to the emergence of antibiotic-resistant isolates worldwide^1^. For example, it has been reported that the prevalence of antibiotic-resistant strains of *Aeromonas hydrophila*, a typical fish pathogen that causes infectious disease outbreaks on farms, is increasing, resulting in huge economic losses^2^. Moreover, the antibiotic resistant strains of this pathogen have also been found in hospitals and municipal wastewaters and have been reported to cause human disease^3^,^4^. Because of the complicated mechanisms of antibiotic resistance, few new drugs are currently being introduced. It is therefore important to investigate antibiotic resistance mechanisms in order to develop new antibiotics and novel antimicrobial targets for the future.

At least 5 principal mechanisms of antibiotic resistance have been well documented. That is, resistance has occurred to drugs that (1) degrade enzymes, (2) bypass target pathways, (3) change antibiotic target sites, (4) alter the permeability of porins, or (5) activate efflux systems^5^,^6^. Despite this, adaptive resistance or “fitness resistance” is a relatively unexplored area that involves a temporary ability to survive antibiotic stress by altering the expression of some genes or proteins—a phenomenon that is arousing increasing interest^7^. For instance, some porins and efflux pumps (e.g., LamB, OmpF, OmpC, and OmpT in *Escherichia coli* and *Serratia marcescens* or the NorA pump in *Staphylococcus aureus*) play a role in adaptive resistance^8^,^9^,^10^. Our previous research demonstrated that global regulatory pathways such as glycolysis/gluconeogenesis, pyruvate metabolism, and the tricarboxylic acid (TCA) cycle may also play important roles in adaptive resistance^11^,^12^,^13^. As a result of studies using metabolomic technologies, both Peng *et al*. and Lobritz *et al*. have pointed out that bacterial metabolic pathways take part in the antibiotics resistance process in both *Staphylococcus aureus* and *E. coli*, indicating a potential strategy for killing bacteria by reversing metabolic pathways^14^,^15^. Until now, however, little research has been done to validate this concept as applied to other bacterial species.

In the present study, we sought to investigate the bacterial adaptive resistance mechanism first by studying the differential expression of *A. hydrophila* under antibiotic oxytetracycline (OXY) stress (OXY being a drug that has been widely used in agriculture). Our results were quantified using label-free and dimethyl labeling-based proteomic technologies. The quantitative results show that several hundred proteins are altered by treatment with varying concentrations of OXY. Except for some well-known antibiotic-related proteins, our bioinformatic analysis also represents the down-regulation of central metabolic pathways involved in the adaptive resistance mechanism. Then, to further evaluate the effect of metabolic pathways on antibiotic resistance in *A. hydrophila*, 32 metabolic substrates were added to disturb the metabolic flows in antibiotics stress. We also found that particular metabolic substrates play important roles in antibiotics resistance. Thus, our study showed that the antibiotic resistance of *A. hydrophila* could potentially be eliminated via applied proteomics and exogenous metabolite assays.

## Results and Discussion

### Label-Free and Dimethyl Labeling Quantitative Proteomic Analysis

**Figure 1:**
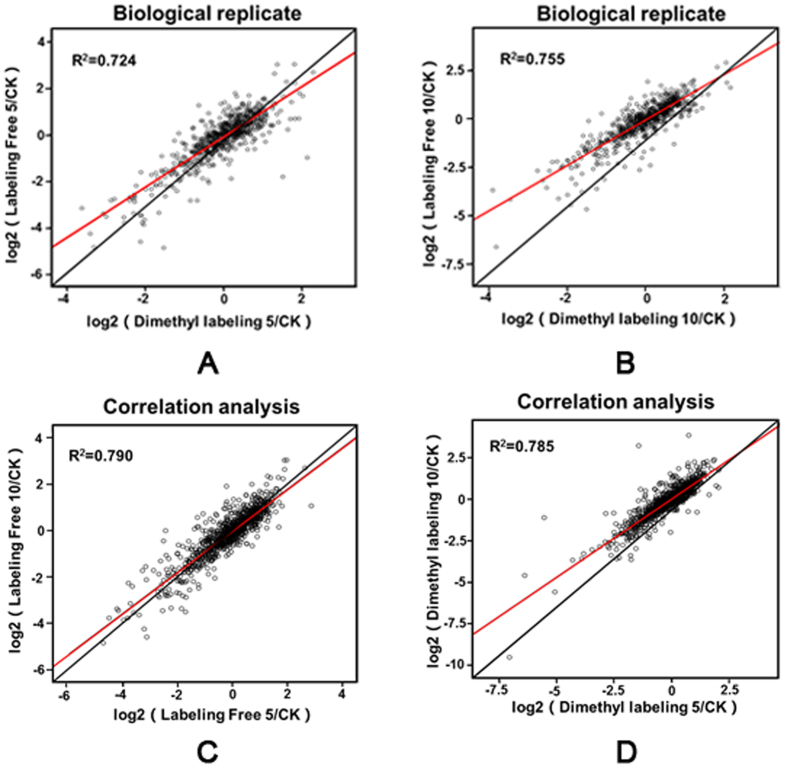
Comparative characteristics of *Aeromonas hydrophila* under different levels of OXY stress. (**A**,**B**) Scatterplots of the biological replicate data between (log_2_ of ratio) the dimethyl labeling and label-free methods in *A. hydrophila* under different OXY treatments of 5- and 10- μg/mL. (**C**,**D**) Correlation analysis of identified proteins in 5- and 10- μg/mL OXY treatments using label-free and dimethyl labeling methods respectively.

To investigate the adaptive resistance mechanism in *A. hydrophila*, two independent group samples were digested in solution to peptides and then either labeled by chemical dimethyl labeling or directly submitted to high-resolution mass spectrometry (MS) for label-free quantification as biological replicates (Supplementary Table Dataset 1). All digested peptides were injected 3 times as technological replicates. Our results showed that all the relative standard deviations of the protein, unique peptide, and peptide groups were lower than 5%, indicating that both methods have excellent repeatability. We compared the correlation of the protein ratios (log_2_ of ratio) between two MS methods under 5- and 10- μg/mL OXY stress and found the R^2^ to be 0.755 and 0.724 respectively, suggesting consistency between both methods (Fig. 1A,B). Because both are well known through proteomic analytic methods and involve different peptide-based quantitative strategies, the combination of these proteomic methods in biological replicates would tend to increase the reliability of our results^16^. We also compared the correlation of the proteins ratios (log_2_ of ratio) under 5- and 10- μg/mL OXY stresses using the same MS method. The results showed that the R^2^ is 0.785 and 0.790 respectively in the two MS methods. This indicates that bacteria behave similarly in responding to the two doses of OXY (Fig. 1C,D).

**Figure 2:**
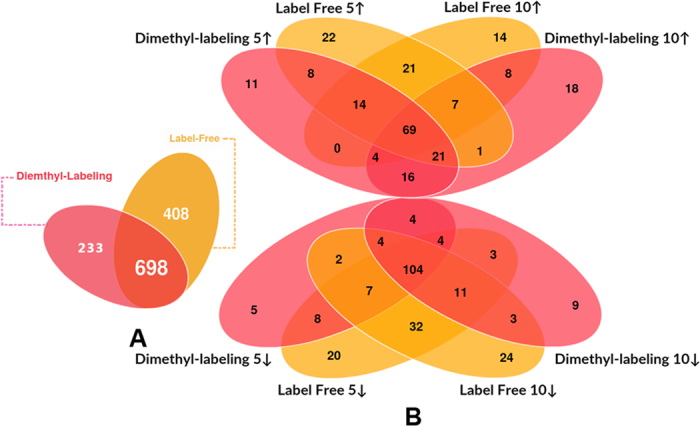
Venn diagrams of overlapping proteins from the comparison between differentially concentrations treatment using 2 quantitative proteomic methods in *Aeromonas hydrophila*. (**A**,**B**) Venn diagrams showing the up- or down- regulated overlap between altered proteins under the treatment of 5- and 10- μg/mL OXY using dimethyl labeling and labeling-free methods, respectively (**B**, on the right). The identification comparison of 2 workflows on *A. hydrophila* is illustrated as a Venn diagram to demonstrate the overlap between dimethyl labeling (pink) and label-free (yellow) methods (**A**, on the left).

Then, using various methods, we looked at the differential expression of *A. hydrophila* proteins after 5- and 10- μg/mL OXY treatments and compared the results with the no-treatment control. In this study, a total of 1,106 proteins including 22,057 unique peptides were identified by the label-free method and 931 proteins including 9,691 unique peptides were identified by dimethyl labeling (Table 1 and Supplementary Dataset 1). Our results showed that 698 proteins identified by the label-free method could also be identified by dimethyl labeling (Fig. 2A). As shown in Fig. 2B, of these commonly identified proteins, a total 352 differential proteins (including 189 decreasing in abundance and 163 increasing in abundance) under 5 μg/mL OXY stress and a total 325 proteins (including 187 decreasing in abundance and 138 increasing in abundance) under 10 μg/mL OXY stress were identified using the label-free method. With the dimethyl labeling method, a total 281 different proteins (including 138 decreasing in abundance and 143 increasing in abundance) under 5 μg/mL OXY stress and 286 proteins (including 142 decreasing in abundance and 144 increasing in abundance) under 10 μg/mL OXY stress were identified. In addition, under 5 μg/mL OXY stress, there are 112 up-regulated proteins and 123 down-regulated proteins overlapped among those identified by the dimethyl labeling and label free methods, while under 10 μg/mL OXY stress, 88 up-regulated and 122 down-regulated overlapped. Furthermore, 69 common proteins were up-regulated under both OXY stress and both MS methods, while 104 were down-regulated. This indicates that these altered proteins may represent stable responses to OXY stress.

### Bioinformatics Analysis Reveals the Translation and Metabolism Pathways Involved in OXY Stress

**Figure 3:**
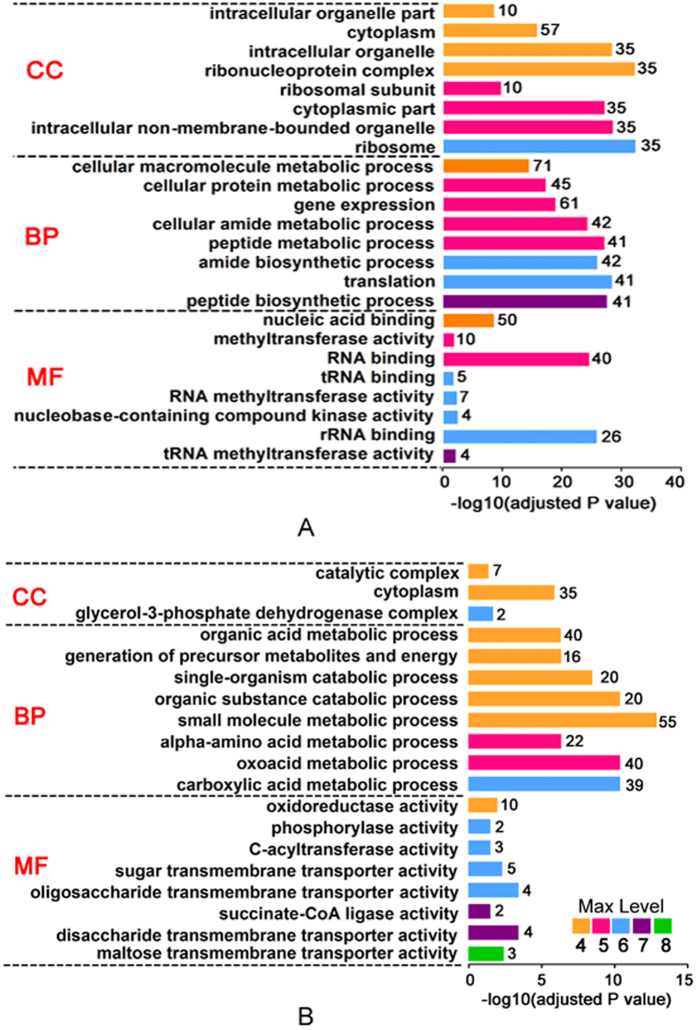
The gene ontology (GO) analysis of differentially expressed proteins in *A. hydrophila* under the treatment of 5 μg/mL OXY. Two charts show the up-regulated (**A**) and down-regulated (**B**) the GO analysis with significantly enriched terms in cell component (CC), biological process (BP) and molecular function (MF) categories at various levels (≥4) respectively. The cutoff of the adjusted *P* value is set at 0.05. Terms of the same category are ordered by *P* values and shown in the y-axis (log_10_ scale on the y-axis). Explanatory information on the functional enrichments and numbers of involved proteins in terms are all listed on the left and right, respectively, behind the bars. Different colors stand for different max levels in the GO analysis from 4 to 8, such as yellow (4), pink (5), blue (6), purple (7), and green (8).

Based on the potential consistency of quantitative results from both methods and both treatments, we took the bacterial behaviors noted under 5 μg/mL OXY stress using the label-free method, for example, to performed GO ontology analysis with differential GO term levels (higher than 3; Fig. 3). According to the GO enrichment analysis based on the cellular component category, the most abundant proteins were located in cytoplasm in whether increased or decreased proteins, whereas 35 ribosome subunits proteins (at level 6) were enriched in increasing-abundance proteins and two glycerol-3-phosphate dehydrogenase complex proteins (glycerol-3-phosphate dehydrogenase and anaerobic glycerol-3-phosphate dehydrogenase subunit A) at the same level were enriched in decreasing-abundance proteins under OXY stress. Recent studies have clearly documented the role of ribosome subunits in antibiotic resistance; this was seen as a potential compensation effect to relieve the burden causing by tetracycline attack^17^. Interestingly, there are many conserved posttranslational modifications, such as lysine acetylation and succinylation modification, in the ribosome subunits, suggesting a novel regulatory mechanism in multiple biological processes including antibiotic resistance^18^,^19^.

It is interesting that cellular macromolecule biosynthetic processes at level 4, including the translational process, were enriched in up-regulated proteins in the biological process category, whereas small molecule metabolic processes—which mostly involve metabolic pathways such as the generation of precursor metabolites and energy—were more enriched in down-regulated proteins. This indicates that there is a significant difference between increasing- and decreasing- abundance proteins in responding to OXY stress. As expected, we found an increasing abundance of translational processes coupled with an increase in multiple binding (such as RNA binding and nucleic acid binding) in the molecular function category. Besides this, we also found 4 proteins (R4VCR0, R4VUQ8, R4VXH8, and R4VCL6) involved in nucleobase- containing compound kinase activity and 7 proteins (R4VBY6, R4VFG9, K1JAM2, R4W2Q0, R4VJZ2, R4VKG0, and R4VSH7) involved in RNA methyltransferase activity in decreasing abundance, suggesting that OXY stress may also have an effect on nucleic acid synthesis. Meanwhile, the decreasing abundance of metabolic process–related proteins resulted in the down-regulation of many catalytic activities. Interestingly, we found that the outer membrane protein LamB and its related proteins (such as MalE, MalK, TreP, and FruA), which play important roles in disaccharide/oligosaccharide and maltose/sugar transmembrane transporter activities, were decreased. In addition, LamB was found to be down-regulated under many antibiotic stresses and in numerous bacterial species. For example, the knockout of this gene in *E. coli* will cause significant resistance to many kinds of antibiotics including tetracycline^8^. Our data indicates that there may be a global regulatory mechanism for bacteria mediated by LamB and related energy metabolic flow in response to antibiotics. Actually, some reports support this opinion, as mentioned above. Using metabolomic methods, researchers have found that metabolic pathways were reversed by the addition of exogenous metabolites and that this increased the susceptibility to antibiotics of *E. coli* and *Staphylococcus aureus*. Our results confirmed this phenomenon in *A. hydrophila* from a proteomics perspective, suggesting a potential new strategy for dealing with this pathogen.

### Western Blotting Validated the Selected Altered Proteins in Proteomics Analysis

**Figure 4:**
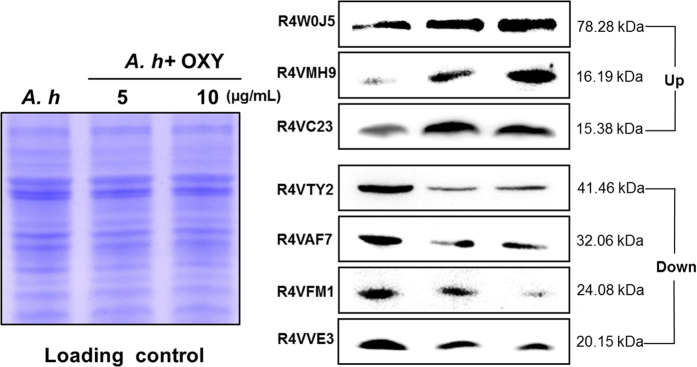
Western blotting analysis of the differentially expressed proteins in *A. hydrophila* under the treatment of 5- and 10- μg/mL OXY. Western blot analysis for R4VC23, R4VAF7, R4VMH9, R4W0J5, R4VFM1, R4VTY2 and R4VVE3 expression in the increasing concentration of OXY. Coomassie staining was used for the loading controls (on the left).

To validate our proteomic data, we selected 7 proteins, 50 S ribosomal protein L9 (R4VC23), an OmpK-like outer membrane protein (R4VAF7), ferric uptake regulation protein (R4VMH9), ferrichrome receptor (R4W0J5), UPF0502 protein(R4VFM1), succinyl-CoA ligase [ADP-forming] subunit beta (R4VTY2) and cytochrome c oxidase subunit 1 (R4VVE3) to measure their expression by Western blotting. We found that the expression of these genes we selected were regulated in different trends under OXY stress. All the genes were cloned, overexpressed of the related proteins and purified to produce specific mouse polyantibodies, respectively. Western blotting showed that these proteins were up- or down- regulated under different concentrations of OXY (Fig. 4). This indicates that our proteomic results appear to be reliable.

### Reverse Metabolic Processes Affect the Susceptibility of *A. hydrophila* to Antibiotics

**Figure 5:**
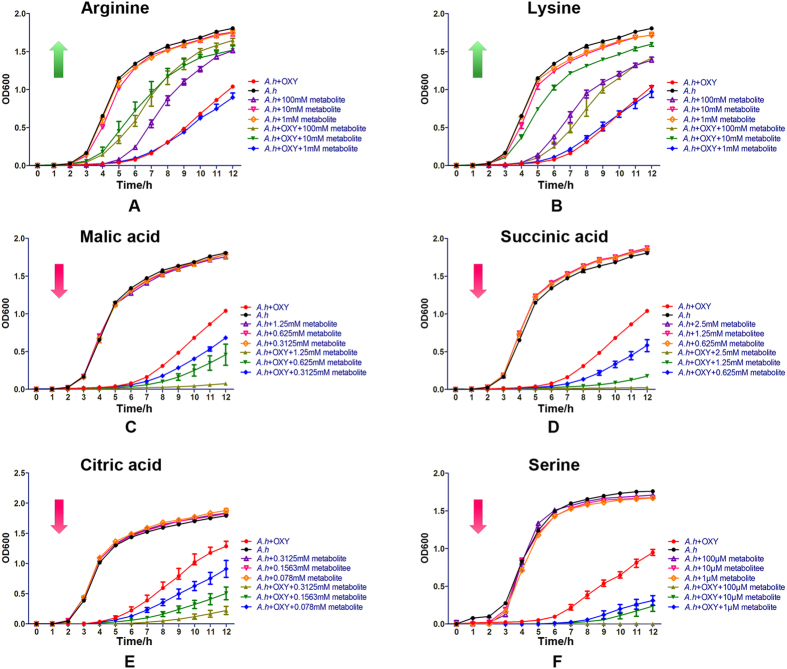
Effect of exogenous metabolites on the susceptibility of *A. hydrophila* to OXY treatment. (**A,B**) The growth curves of *A. hydrophila*, which were treated with or without 0.6 μg/mL OXY and reversed exogenous metabolites (arginine, lysine). The concentrations of these reagents are in the gradient of 0, 1, 10, and 100 mM. (**C** to **F**) The other growth curves of *A. hydrophila* were treated with or without the final concentration of 0.6 μg/mL OXY and inhibited exogenous metabolites (malic acid in the gradient of 0, 0.3125, 0.625, and 1.25 mM; succinic acid in the gradient of 0, 0.625, 1.25, and 2.5 mM; citric acid in the gradient of 0, 0.078, 0.1563, and 0.3126 mM, and serine in the gradient of 0, 1, 10, and 100 mM). All growth kinetics of treated cells were recorded by absorbance measurements of OD_600_ nm at 30 °C with the multimode detection platform every hour for 12 hours.

**Figure 6:**
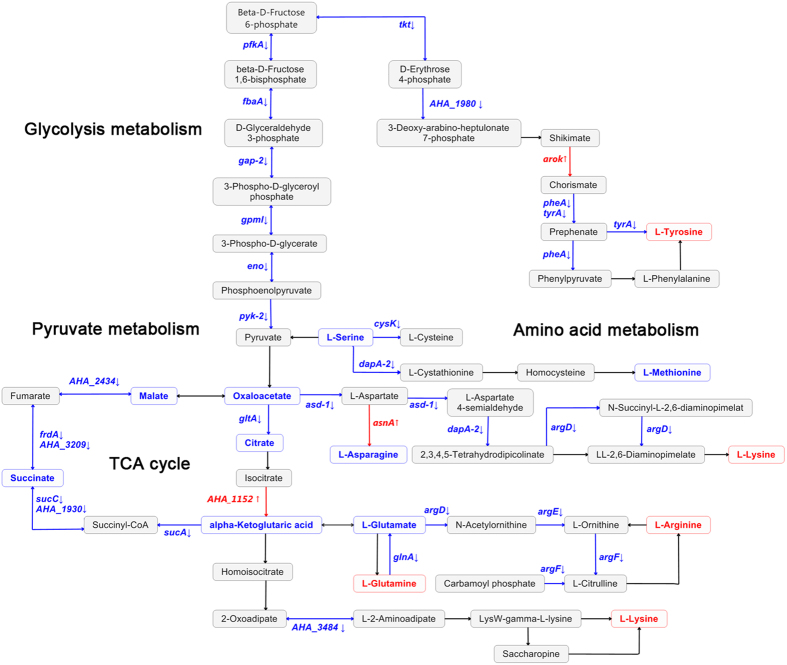
Schematic presentation of the glycolysis mechanism, pyruvate metabolism, and amino acid metabolism for OXY treatment in *A. hydrophila.* The identified up-regulated proteins (in red) and down-regulated proteins (in blue) are all shown above the arrow in the flowchart. According to the Kyoto Encyclopedia of Genes and Genomes (KEGG) database, the exogenous metabolites lowered the survival capabilities of *A. hydrophila* in the final concentration 0.6 μg/mL OXY (i.e., malate, succinate, citrate, glutamate, asparagine, serine, oxaloacetate, α-ketoglutaric acid, and methionine) (in blue). The other 4 metabolites (i.e., arginine, lysine, tyrosine, and glutamine) (in red) significantly reversed its survival capabilities.

Our proteomic results showed a decrease in the central metabolic pathways of *A. hydrophila* under the influence of OXY, which indicates that we may reverse antibiotics resistance properties by controlling bacterial metabolic flows, as previously reported in other bacterial species. To validate this 32 exogenous metabolites covering a large range of central metabolic pathways (such as the TCA cycle, pyruvate metabolic, glycogenesis/glycogenolysis metabolism and amino acid metabolism) were added to LB medium with a serial OXY concentration. The survival capabilities of *A. hydrophila* subjected to the different doses of the metabolites and OXY compounds were measured with their separate treatments as control. We choose a modest OXY (0.6 μg/mL) concentration as a treatment dose and then treated the pathogen with serial metabolite concentrations. As shown in Fig. 5, the effective supply of exogenous metabolites could be roughly divided into 2 categories. Seven metabolites (malic acid, succinic acid, citric acid, glutamic acid, asparagine, serine, and methionine) significantly decreased the survival capabilities of *A. hydrophila* in constant 0.6 μg/mL OXY. However, addition of metabolites alone lead to no significant effect on bacterial survival except high dose of treatment. Taking exogenous malic acid as an example, the growth was drastically depressed under the treatment of 1.25 mM of exogenous malic acid together with OXY added. On the other hand, the survival capabilities of *A. hydrophila* were significantly reversed compared with control when 4 metabolites (arginine, lysine, tyrosine, and glutamine) were added. Exogenous lysine alleviated the pressure from OXY stimulation at a concentration of 10 mM. Our exogenous metabolite experiments indicate that metabolic pathways play a role in the bacterial resistance mechanism and that the appropriate regulation of these metabolic flows can help to kill bacteria when metabolites are compounded with antibiotics. Of these 7 exogenous metabolites that effectively inhibited bacterial growth with OXY compound, malic acid, succinic acid, and citric acid are key products in the TCA cycle, which is consistent with our proteomic results and indicates the important role of central metabolic process in antibiotics resistance (Fig. 6). Various exogenous amino acid metabolisms may also affect metabolic flows and lead to fluctuations in antibiotic susceptibilities.

### Exogenous Metabolites and Antibiotic Compounds Affect Bacterial Susceptibility to Antibiotics

**Figure 7:**
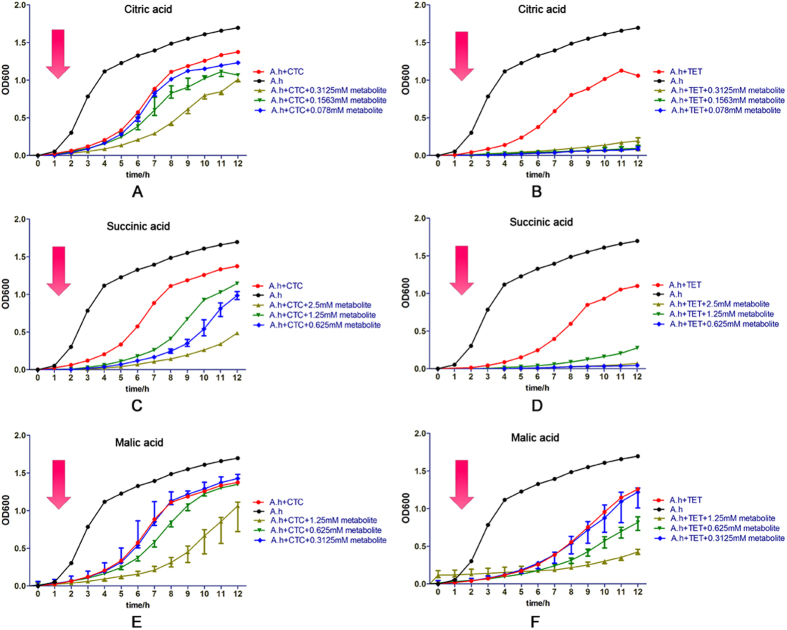
Selected exogenous metabolites compounded with different antibiotics affect bacterial growth. (**A** to **F**) The growth curves of *A. hydrophila* after treatment with tetracyclines by adding 0.5 μg/mL TET and 5 μg/mL CTC with different exogenous metabolites (i.e., citric acid in the gradient of 0, 0.078, 0.1563, and 0.3126 mM; succinic acid in the gradient of 0, 0.625, 1.25, and 2.5 mM; and malic acid in the gradient of 0, 0.3125, 0.625, and 1.25 mM). All growth kinetics of treated cells were recorded every hour for 12 hours by absorbance measurements at OD_600_ nm and 30 °C with the multimode detection platform. Abbreviations: tetracycline (TET), chlortetracycline (CTC).

To further determine whether the behavior of bacteria, in response to metabolites and antibiotic compounds, is OXY-specific or not, we measured the survival rates of *A. hydrophila* strain. The strain was treated with the three most effective bactericidal metabolites (citric acid, malic acid, and succinic acid) found in our study together with other compounds that belong to tetracyclines, quinolones, penicillins, chloramphenicols, aminoglycosides, and nitrofurans. As Fig. 7 and Supplementary Table S1 show, we first found that tetracycline antibiotics such as chlortetracycline (CTC) and tetracycline (TET) compounded with the 3 exogenous metabolites significantly decreased the survival of *A. hydrophila*, as did the OXY compound (Fig. 7). This indicates that exogenous metabolites would affect the bacterial susceptibility to tetracyclines. Besides these, a nalidixic acid (NAL, quinolones) compound with citric acid and succinic acid, respectively; an ampicillin (AMP, penicillins) compound with malic acid and succinic acid, respectively; chloramphenicol (CHL, chloramphenicols) with citric acid; and furazolidone (FUR, nitrofurans) with succinic acid significantly increased antibiotic susceptibilities as well (Supplementary Fig. S2). Interestingly, we also found that some antibiotics belonging to the aminoglycosides could increase susceptibility or have a greater tendency to do so when the 3 exogenous metabolites were compound with kanamycin (KAN) and spectinomycin (SD) (Supplementary Fig. S3). In addition, the antibiotic susceptibility of *Edwardsiella tarda* to kanamycin was reported to be affected by the exogenous metabolite fructose compounded with kanamycin^20^. This result may stem from unique antibiotic resistance mechanisms related to specific antibiotics. In general, many exogenous metabolites compounded with antibiotics may serve as potential bactericidal strategies and therefore require further investigation.

## Conclusions

Although we know little about its intrinsic mechanisms, bacterial adaptive resistance is an attractive field because it could inspire intracellular antibiotic resistance in a short period of time without genetic mutations. In this study, using two different quantitative proteomic methods, we compared the differential expression of *A. hydrophila* under OXY stress. Further bioinformatics analysis and selected altered proteins subjected to Western blotting verified that translation-related proteins increased while many central metabolic-related proteins decreased under OXY stress. On the basis of the key metabolic processes that play important roles in intracellular physiological homeostasis and are involved in a variety of complex biological processes, we assume that these metabolic flows should participate in bacterial adaptive resistance. Thus we screened numerous exogenous metabolites that are involved in various metabolic pathways to find if the antibiotics susceptibilities are affected if the metabolic flows are altered. Our results show that antibiotic susceptibility can be significantly inhibited when such metabolites are compound with OXY antibiotics. Thus our findings may provide novel clues for antimicrobial therapy in *A. hydrophila* infection.

## Methods

### Bacterial Strains and Sample Preparation

The bacterial strains and plasmids used in this study are described in Table 1. The adaptation of bacterial antibiotics was conducted by the inclusion of OXY, as previously described, with small modifications^21^. Briefly, a single colony of *A. hydrophila* ATCC 7966 was propagated in 5 mL of LB medium at 37 °C with shaking at 200 rpm. The overnight culture was diluted 1:100 to fresh LB medium in the same incubation condition for about 3 hours to reach the midlogarithmic phase (OD_600_ = 0.6~1.0). OXY at final concentrations of 5- and 10- μg/mL respectively was added to the medium for 1 hour; the cells were then harvested. After being washed with PBS buffer twice, the cells were disrupted by intermittent sonic oscillation for a total of 25 minutes at 9 seconds intervals on ice and then centrifuged at 5000 g for 10 minutes to remove cellular debris. Protein pellets were solubilized in digestion buffer (8 M urea, 0.1% SDS, and 2% Triton X-100 in 0.5 M triethyl ammonium bicarbonate buffer, pH 8.5); thereafter the total protein concentration was estimated using Bradford assay for immediate use; otherwise the solution was stored at −80 °C.

### In-Solution Digestion and Stable Isotope Dimethyl Labeling

Briefly, about 100 μg of protein for each group was reduced by DL-Dithiothreitol (DTT) at 37 °C for 1 hour, alkylated by Iodoacetamide (IAA) at room temperature in the dark for 1 hour, and digested by trypsin in a ratio of 1: 50. It was then stored at 37 °C overnight, as in our previous study^22^. The digested peptides were labeled using the on-column stable isotope dimethyl labeling procedure as previously described or directly submitted to mass spectrometry for label-free evaluation^23^. The labeling scheme was *A. hydrophila* in LB medium without OXY, with 5 μg/mL OXY, and with 10 μg/mL OXY. These had light, intermediate, and heavy dimethyl labels. The pooled labeled or directly digested peptides were desalted using a Sep-Pak Vac C18 cartridge 1 mL/100 mg (Waters Inc., Milford, MA) and then dried using a CentriVap Concentrator (Labconco Inc., Kansas City, MO).

### LC MS/MS

Dried peptides were resuspended in 0.1% formic acid (FA) and then analyzed by liquid chromatography–tandem mass spectrometry (LC-MS/MS) analysis in a Q-Exactive mass spectrometer (Thermo Fisher Scientific, San Jose, CA) with Thermo Scientific Easy-nLC 1000 system. Briefly, peptides were loaded on the Easy-spray column (C18, 2 μm, 100 Å, 75 μm x 50 cm) with a 180-minute 3% to 90% acetonitrile/water gradient containing 0.1% FA at a constant flow rate of 250 nL/min. The solvents were buffer A (0.1% FA in water) and buffer B (0.1% FA in acetonitrile) under following gradient elution: 3% to 8% B from 0 to 5 minutes, 8% to 20% B from 5 to 140 minutes, 20% to 30% B from 140 to 165 minutes, 30% to 90% B from 165 to 170 minutes, and 90% B from 170 to 180 minutes. The ion spray voltage was set at 2.3 kV in the positive ion mode. All ions were operated with an ion source temperature of 270 °C and S-lens setting of 55%; A data-dependent top-20 method was used, dynamically choosing the most abundant precursor ions. For the compounds of interest, a scan range of 300 to 1800 m/z was chosen and MS/MS was acquired at 100 m/z. Normalized high-energy collision dissociation (HCD) energy was set at 27% and the resolution for HCD spectra was set to 17,500 at m/z 200 on the Q Exactive mass spectrometer.

Raw mass spectrometric data were analyzed with using the MaxQuant pipeline as previously described^24^. The fragmentation spectra were searched against the *A. hydrophila* ATCC 7966 database (download from Uniport database on 1/1/2016). The search parameters including alkylated cysteine residue by IAA, trypsin digestion. The additional modification is dimethyl for dimethyl labeling. For label-free procedures, protein abundance quantification was carried out on the identified peptides using the LFQ algorithm in MaxQuant^24^. Each sample was repeated 3 times as technological replicates. The protein quantitation and confidence were performed using a relatively conservative threshold (folds change ≥ 1.5 or ≤0.667, false discovery rate <1% with at least 2 peptides matched were considered for further analysis). Each experiment was performed in at least triplicate as replicates and was normalized with related peptides of control.

### Gene Ontology Categories and Bioinformatics Analysis

The overlapping proteins of altered proteins among those proteomic methods and two OXY treatments were analyzed using online Venny tool in a Venn diagram (http://creately.com/Draw-Venn-Diagrams-Online). The gene ontology (GO) terms of differential proteins in this study were analyzed using online software Omics Bean (http://www.omicsbean.com), which is a new powerful multiomics data approach for obtaining proteomics data analysis^25^. Distributions of cell component (CC), biological process (BP), and molecular function (MF) were enriched for the QuickGO dataset (http://www.ebi.ac.uk/QuickGO/). The cutoff *P* value is set to 0.05 and all terms with a *P* value < 0.05 were considered statistically significant.

### Validation of Proteomic Assays

To further verify the altered proteins in OXY stress, the genes from randomly selected altered proteins were cloned. The targeted proteins were purified and then immunized mouse to produce specific polyclonal antibodies as previously described^26^. The total bacterial proteins were prepared and separated by 12% SDS-PAGE and then transferred to the polyvinylidene fluoride (PVDF) membrane for half an hour at 1.3 A in transfer buffer (Bio-Rad, 1X Tris/Glycine Buffer) at room temperature (RT). The membranes were washed in PBST buffer for 5 minutes and blocked in PBST containing 5% (w/v) skim milk and incubated for 1 hour at RT with a mouse antisera to R4VC23 (50 S ribosomal protein), R4VAF7 (outer membrane protein), R4VMH9 (ferric uptake regulation protein), R4W0J5 (ferrichrome receptor), R4VFM1 (UPF0502 protein), R4VTY2 (succinyl-CoA ligase [ADP-forming] subunit beta) and R4VVE3 (cytochrome c oxidase subunit 1) at 1: 3000 dilutions as the primary antibody at 4 °C for 12 hours. After washing 3 times for 5 minutes each time with PBST buffer the membranes were stained with horseradish peroxidase conjugated goat antimouse antibody at 1: 4000 dilutions as the secondary antibody in PBST buffer with 3% (w/v) skim milk for incubating an hour at RT. The immunostained proteins were visualization by Clarity™ Western ECL Substrate (Bio-Rad, Hercules, CA) and recorded by ChemiDoc MP imaging system with Image Lab software (Bio-Rad, Hercules, CA). The PVDF membranes were further stained by coomassie R-350.

### *In Vivo* Exogenous Metabolite Addition Assay

For the *in vivo* exogenous metabolite stimulation assay, the multivariate growth curves were measured and the exogenous metabolites added as previously described^20^. *A. hydrophila* was diluted at 1: 100 in an appropriate dose of antibiotics with serials of concentrations of a total 32 exogenous metabolites including malic acid, succinic acid, citric acid, glutamic acid, asparagine, serine, methionine, oxaloacetic acid, α-ketoglutaric acid, arginine, lysine, tyrosine, and glutamine ect. treatments at 30 °C with shaking at 200 rpm, respectively. The cell densities were determined for 12 hours via optical density (OD) measurement at 600 nm using the Bioscreen C system (Labsystems, Helsinki, Finland). All experiments were performed in triplicate and GraphPad Prism version 5.0 was used for statistical analyses.

## Additional Information

**How to cite this article:** Yao, Z. *et al*. Proteomic Analysis Reveals That Metabolic Flows Affect the Susceptibility of *Aeromonas hydrophila* to Antibiotics. *Sci. Rep.* **6**, 39413; doi: 10.1038/srep39413 (2016).

**Publisher's note:** Springer Nature remains neutral with regard to jurisdictional claims in published maps and institutional affiliations.

## Supplementary Material

Supplementary Information

Supplementary Dataset 1

## Figures and Tables

**Table 1:** Selected altered proteins of *Aeromonas hydrophila* with 5- and 10- μg/mL oxytetracycline stress using Label-Free and Dimethyl labeling Methods.

Protein IDs	Description	Unique Peptides	Sequence Coverage [%]	5 Dim	10 Dim	5 Free	10 Free
R4VWN4	Transporter	4	11.8	4.50	3.84	3.00	3.46
R4VR16	Import inner membrane translocase subunit Tim44	2	7	4.22	3.56	3.58	4.64
R4VL01	UDP-3-O-[3-hydroxymyristoyl] N-acetylglucosamine deacetylase	4	19.9	4.16	1.61	7.31	2.07
R4VXJ1	Uncharacterized protein	5	38.2	3.55	4.92	6.24	6.41
R4VY09	Ribosome-binding factor A	8	56.2	3.03	4.08	2.36	3.17
R4VXN9	Peptidyl-tRNA hydrolase	5	30.8	2.72	2.67	1.44	1.44
R4VJZ2	tRNA (guanine-N(7)-)-methyltransferase	3	21.1	2.68	2.76	2.33	2.03
R4VR84	Glycerol-3-phosphate acyltransferase	8	14.7	2.47	2.02	1.30	0.99
R4VBY6	tRNA (guanine-N(1)-)-methyltransferase	4	16.5	2.45	2.89	2.69	3.14
R4VTV5	Protease	7	18.3	2.42	2.06	1.64	1.52
A0KKZ5	Phosphorylase	28	40.7	0.21	0.24	0.06	0.09
K1JCQ9	Uncharacterized protein	3	37	0.21	0.32	0.21	0.10
R4VQF5	Aspartate ammonia-lyase	18	39.6	0.19	0.21	0.21	0.22
A0KHF6	Maltoporin	10	42.7	0.18	0.21	0.15	0.16
R4VMV4	Maltose/maltodextrin import ATP-binding protein MalK	10	36	0.17	0.17	0.08	0.09
R4VYS5	Bifunctional acetaldehyde-CoA/alcohol dehydrogenase	35	50.1	0.16	0.10	0.11	0.05
R4VW15	Maltose ABC transporter periplasmic protein	23	62.1	0.15	0.14	0.15	0.14
K1JGL1	Uncharacterized protein	2	47.8	0.09	0.12	0.05	0.10
K1JJ73	Ornithine carbamoyltransferase	16	56.6	0.07	0.10	0.01	0.04
R4VET1	Ribosomal subunit interface protein	5	45.5	0.07	0.08	0.08	0.11

NOTE: 5 Dim and 10 Dim mean altered proteins using the dimethyl labeling method in 5- and 10- μg/mL OXY stress respectively; 5 Free and 10 Free mean altered proteins using label-free method in 5- and 10- μg/mL OXY stress respectively.
