# Supplementary material for: Proteomic Analysis Reveals That Metabolic Flows Affect the Susceptibility of Aeromonas hydrophila to Antibiotics
Source: Sci Rep. 2016 Dec 19;6:39413. doi: 10.1038/srep39413 (PMC5171847; doi:10.1038/srep39413)
Supplement: Supplementary Information [file srep39413-s1.doc]

Proteomic Analysis Reveals That Metabolic Flows Affect the Susceptibility of *Aeromonas hydrophila* to Antibiotics

Zujie Yao ^1,2^ , Wanxin Li ^1,2^, Yi Lin^3^, Qian Wu^1,2^, Feifei Yu^1,2^, Wenxiong Lin^1,2#^ & Xiangmin Lin^1,2*#^

^1^ Fujian Provincial Key Laboratory of Agroecological Processing and Safety Monitoring, College of Life Sciences, Fujian Agriculture and Forestry University, Fuzhou 350002, PR China

^2^ Key Laboratory of Crop Ecology and Molecular Physiology of Fujian Universities, Fujian Agriculture and Forestry University, Fuzhou 350002, PR China

^3^ Nanping Enter-Exit Inspection and Quarantine Bureau, Nanping 353000, PR China

#Corresponding author: Xiangmin Lin, E-mail: xiangmin@fafu.edu.cn and Wenxiong Lin, E-mail: lwx@fjau.edu.cn, Agroecological Institute, Fujian Agriculture and Forestry University, Fuzhou 350002, Fujian, PR China. Tel: +86059183769440; Fax: +86059183769440;

**Supplementary figure and table legends**

**Figure S1. Effect of other exogenous metabolites on the susceptibility of *A. hydrophila* to OXY treatment.** (A and B) The growth curves of *A. hydrophila*, which were treated with or without 0.6 µg/mL OXY and reversed exogenous metabolites (tyrosine in the gradient of 0, 0.02, 0.2, and 2 mM and glutamine in the gradient of 0, 0.2, 2, and 20 mM). (C to H) The other growth curves of *A. hydrophila* were treated with or without the final concentration of 0.6 µg/mL OXY and inhibited exogenous metabolites (methionine in the gradient of 0, 0.2, 2, and 20 mM; glutamic acid in the gradient of 0, 0.02, 0.2, and 2 mM; asparagine in the gradient of 0, 0.2, 2, and 20 mM; α-ketoglutaric acid in the gradient of 0, 1, 10, and 100 mM; oxalic acid in the gradient of 0, 1, 10, and 100 mM; and oxaloacetate in the gradient of 0, 1, 10, and 100 mM). All growth kinetics of treated cells were recorded by absorbance measurements at OD_600_ nm and 30°C with the multimode detection platform every hour for 12 hours.

**Figure S2. Selected exogenous metabolites compounded with different antibiotics affect bacterial growth.** (A to F) The growth curves of *A. hydrophila* after treatment with antibiotics by adding 0.1 µg/mL NAL, 0.5 µg/mL CHL, 200 µg/mL AMP, and 1 µg/mL FUR with different exogenous metabolites (i.e., citric acid in the gradient of 0, 0.078, 0.1563, and 0.3126 mM; succinic acid in the gradient of 0, 0.625, 1.25, and 2.5 mM; and malic acid in the gradient of 0, 0.3125, 0.625, and 1.25 mM). All growth kinetics of treated cells were recorded by absorbance measurements at OD_600_ nm and 30 °C with the multimode detection platform every hour for 12 hours. Abbreviations: nalidixic acid (NAL), chloramphenicol (CHL), ampicillin (AMP), furazolidone (FUR).

**Figure S3. Selected exogenous metabolites compounded with different antibiotics affect bacterial growth.** (A to I) The growth curves of *A. hydrophila* after treatment with aminoglycosides by adding 5 µg/mL KAN, 30 µg/mL SD, 5 µg/mL SM, and 1 µg/mL FUR with different exogenous metabolites (i.e., citric acid in the gradient of 0, 0.078, 0.1563, and 0.3126 mM; succinic acid in the gradient of 0, 0.625, 1.25, and 2.5 mM; and malic acid in the gradient of 0, 0.3125, 0.625, and 1.25 mM). All growth kinetics of treated cells were recorded by absorbance measurements at OD_600_ nm and 30°C with the multimode detection platform every hour for 12 hours. Abbreviations: kanamycin (KAN), spectinomycin (SD), streptomycin (SM).

**Supplementary Table S1. Results from the addition of exogenous metabolites**

**Figure S1.**


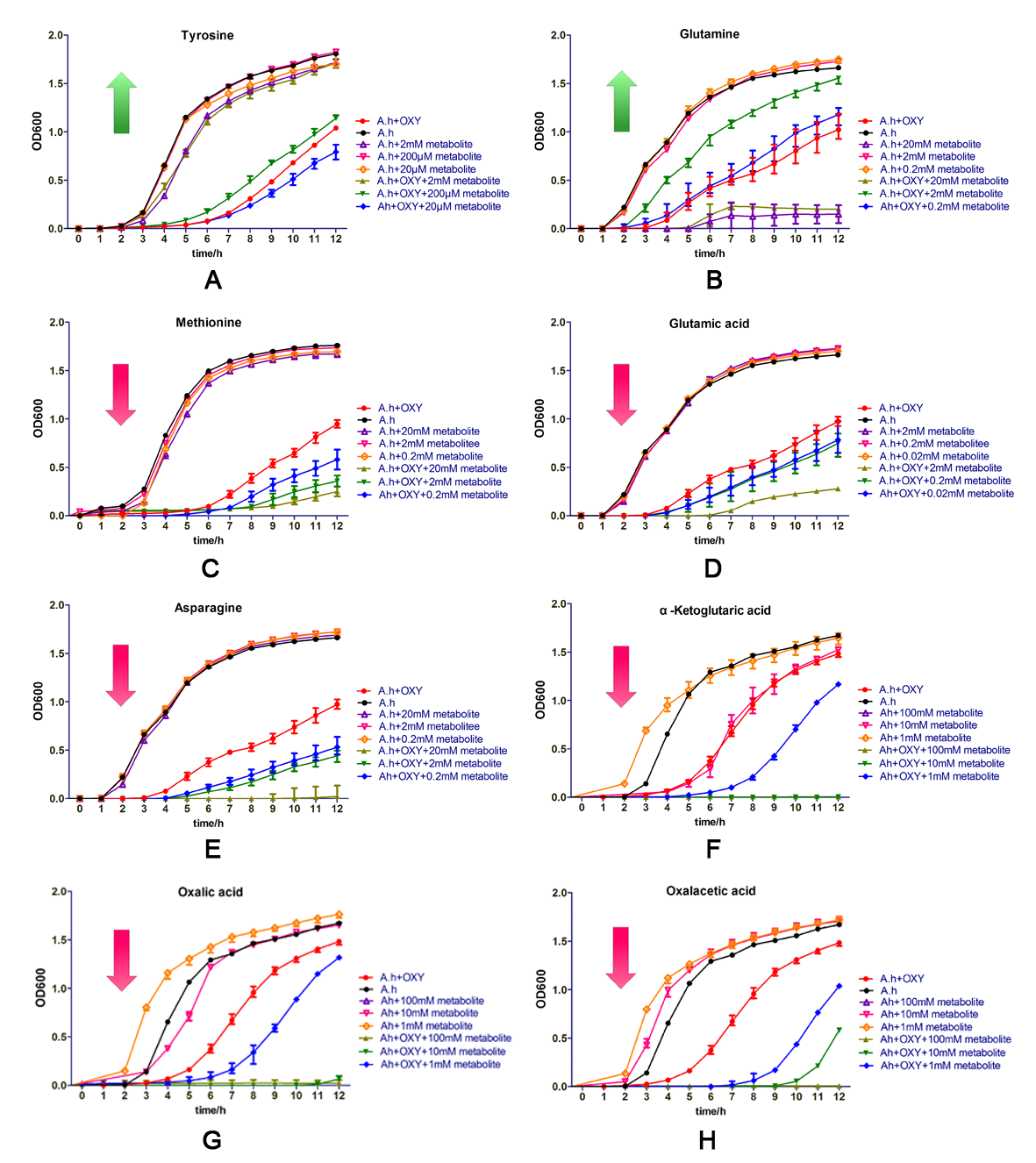


**Figure S2.**


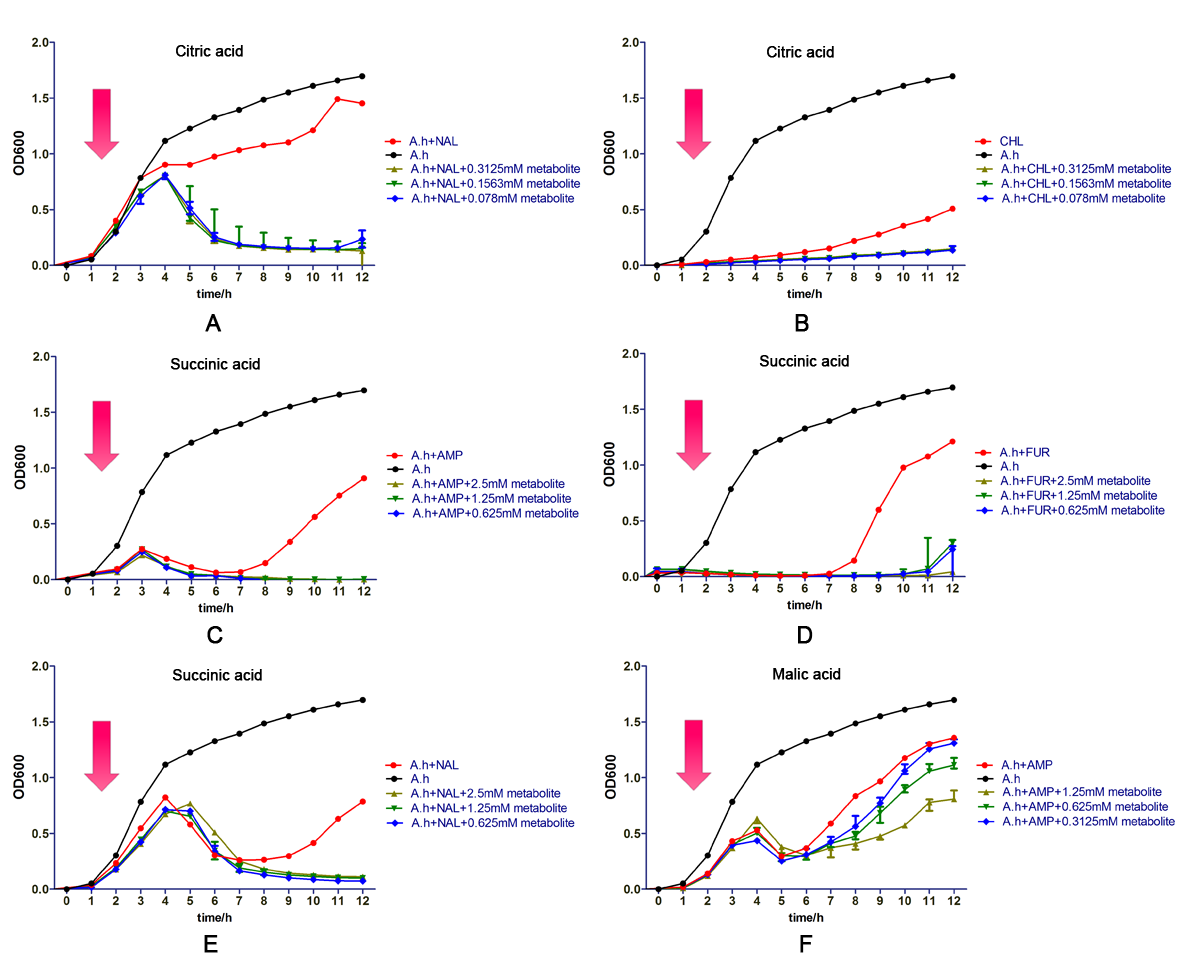


**Figure S3.**


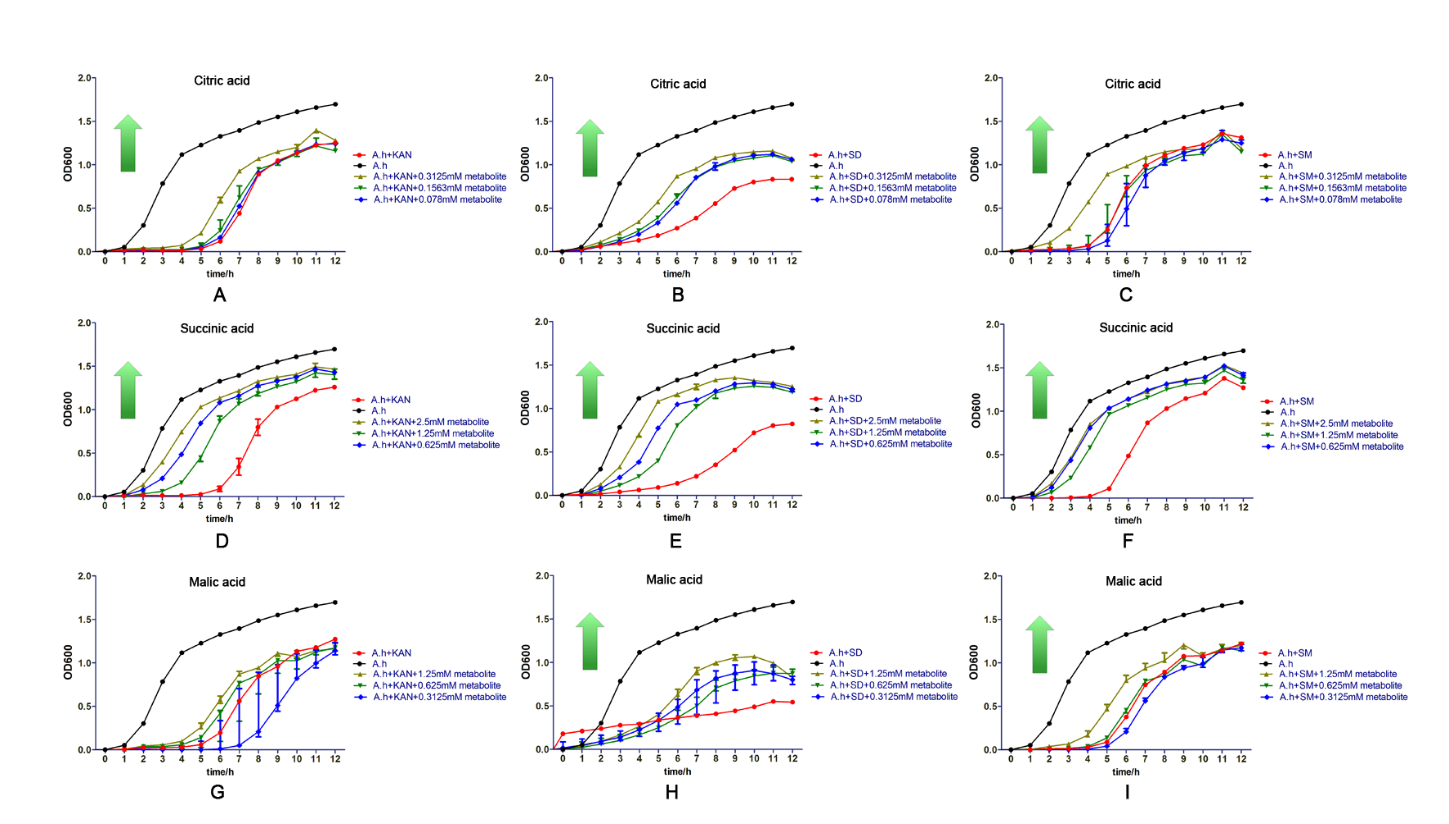


**Supplementary Table S1**. Results from the addition of exogenous metabolites

| **Antibiotics** | **OXY** | **TET** | **CTC** | **NAL** | **CHL** | **AMP** | **FUR** | **KAN** | **SD** | **SM** | **CEF** |
| --- | --- | --- | --- | --- | --- | --- | --- | --- | --- | --- | --- |
| **Citric acid** | ↓ | ↓ | ↓ | ↓ | ↓ | - | - | ↑ | ↑ | ↑ | - |
| **Malic acid** | ↓ | ↓ | ↓ | - | - | ↓ | - | ↑ | ↑ | ↑ | - |
| **Succinic acid** | ↓ | ↓ | ↓ | ↓ | ↓ | ↓ | ↓ | ↑ | ↑ | ↑ | - |
| **Glutamic acid** | ↓ | N | N | N | N | N | N | N | N | N | N |
| **Asparagine** | ↓ | N | N | N | N | N | N | N | N | N | N |
| **Serine** | ↓ | N | N | N | N | N | N | N | N | N | N |
| **Methionine** | ↓ | N | N | N | N | N | N | N | N | N | N |
| **Arginine** | ↑ | N | N | N | N | N | N | N | N | N | N |
| **Lysine** | ↑ | N | N | N | N | N | N | N | N | N | N |
| **Glutamine** | ↑ | N | N | N | N | N | N | N | N | N | N |
| **Tyrosine** | ↑↓ | N | N | N | N | N | N | N | N | N | N |
| **α-Ketoglutaric acid** | ↓ | N | N | N | N | N | N | N | N | N | N |
| **Oxaloacetic acid** | ↓ | N | N | N | N | N | N | N | N | N | N |
| **Propanedioic acid** | ↓ | N | N | N | N | N | N | N | N | N | N |
| **Oxalic acid** | ↓ | N | N | N | N | N | N | N | N | N | N |
| **Glucose** | ↓ | N | N | N | N | N | N | N | N | N | N |
| **Sucrose** | ↓ | N | N | N | N | N | N | N | N | N | N |
| **D-Froctose** | ↓ | N | N | N | N | N | N | N | N | N | N |
| **Glycine** | – | N | N | N | N | N | N | N | N | N | N |
| **Isoleucine** | – | N | N | N | N | N | N | N | N | N | N |
| **Proline** | – | N | N | N | N | N | N | N | N | N | N |
| **Cysteine** | – | N | N | N | N | N | N | N | N | N | N |
| **Guanine** | – | N | N | N | N | N | N | N | N | N | N |
| **Valine** | – | N | N | N | N | N | N | N | N | N | N |
| **Cytosine** | – | N | N | N | N | N | N | N | N | N | N |
| **Alanine** | – | N | N | N | N | N | N | N | N | N | N |
| **Tryptophan** | – | N | N | N | N | N | N | N | N | N | N |
| **Leucine** | – | N | N | N | N | N | N | N | N | N | N |
| **Aspartic acid** | – | N | N | N | N | N | N | N | N | N | N |
| **Histidine** | – | N | N | N | N | N | N | N | N | N | N |
| **Uridine** | – | N | N | N | N | N | N | N | N | N | N |
| **Hypoxanthine** | – | N | N | N | N | N | N | N | N | N | N |

NOTE: ↓means that antibiotic susceptibility is decreased; ↑ means that antibiotic susceptibility is increased; ↑↓ means that antibiotic susceptibility increased with higher-dose antibiotics and decreased with lower-dose antibiotics; – means no significant change; N means assay not performed. FUR, furazolidone; AMP, ampicillin; OXY, oxytetracycline; KAN, kanamycin; TET, tetracycline; CTC, chlortetracycline; NAL, nalidixic acid; CHL, chloramphenicol; SD, spectinomycin; SM, streptomycin; CEF, cefotaxime sodium salt; FUR, furazolidone.
